# Supplementary material for: Time to recovery of neonatal sepsis and determinant factors among neonates admitted in Public Hospitals of Central Gondar Zone, Northwest Ethiopia, 2021
Source: PLoS One. 2022 Jul 28;17(7):e0271997. doi: 10.1371/journal.pone.0271997 (PMC9374017; doi:10.1371/journal.pone.0271997)
Supplement: S1 File — (PDF) [file pone.0271997.s001.pdf]

Supplementary file 1: Proportional allocation of each hospital

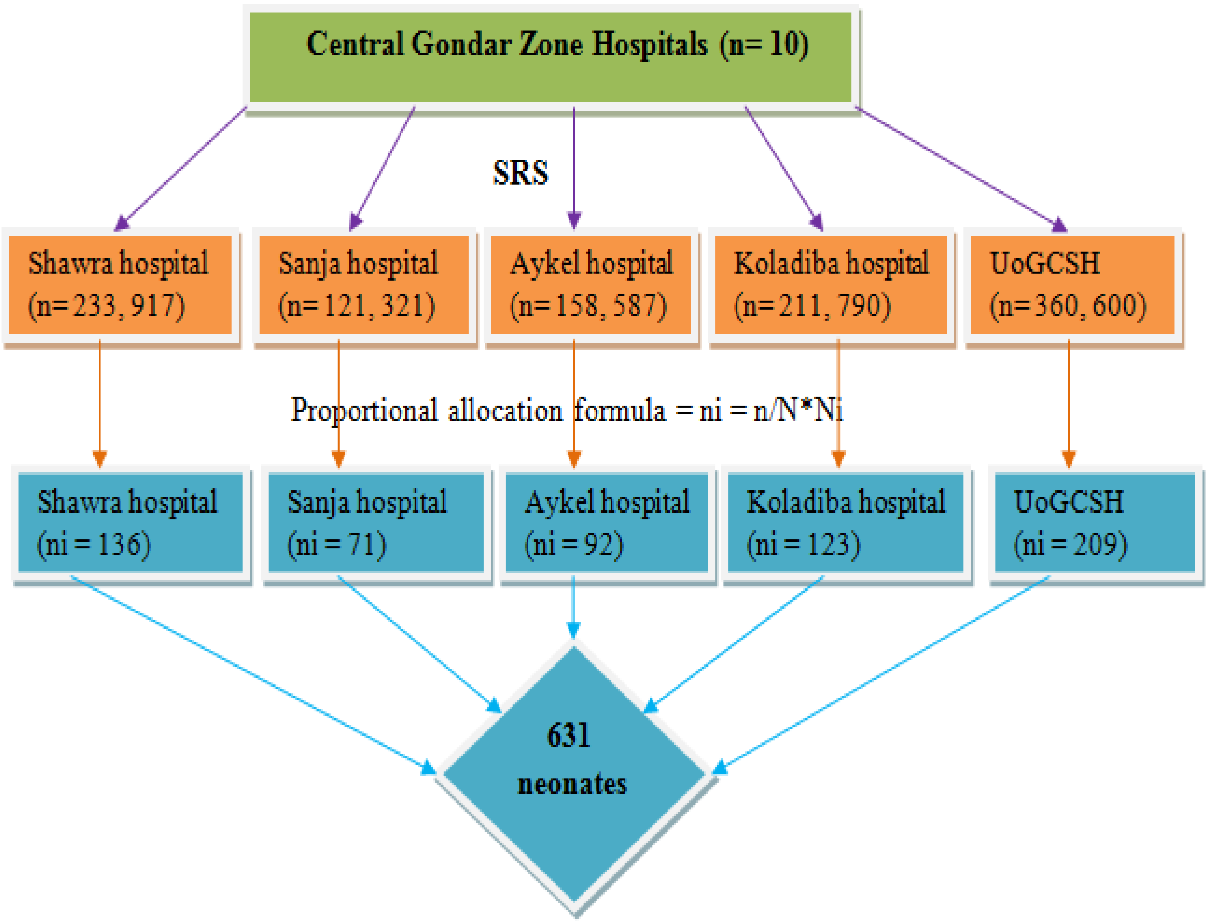

S1_File: Schematic representation of sampling technique with proportional allocation of sample size in each selected public hospital, Central Gondar Zone, Northwest Ethiopia, 2021.

Key: UoGCSH= University of Gondar Comprehensive Specialized Hospital, SRS= Simple random sampling. ni is sample size ith stratum, n is a total sample size, N is a total population size, and Ni is population size of ith stratum.
