# Supplementary material for: Time to recovery of neonatal sepsis and determinant factors among neonates admitted in Public Hospitals of Central Gondar Zone, Northwest Ethiopia, 2021
Source: PLoS One. 2022 Jul 28;17(7):e0271997. doi: 10.1371/journal.pone.0271997 (PMC9374017; doi:10.1371/journal.pone.0271997)
Supplement: S2 File — (PDF) [file pone.0271997.s002.pdf]

Supporting information 2

Interviewer administered questionnaire was prepared to assess the time to recovery of neonatal sepsis and determinant factors among neonates admitted in Public Hospitals of Central Gondar Zone, Northwest Ethiopia, 2021

The name of the institution/hospital ___________________________________

Annex 1: Introduction

A study on the time to recovery of neonatal sepsis and determinant factors among neonates

Unique card number ___________

Date of interview ________

Time at the beginning of the interview_____

My name is ___________________.

Currently, we are working on research that tries to address about survival status of neonates with sepsis and its determinant factors. This improves the clinical evaluation and gives useful information that can be used to follow the health status of the neonates with sepsis and to early recognize and intervene the factors that affect the survival status of the neonates. You and your neonate have been chosen for the study.

Annex 2: Information sheet

Title of the research study: Time to recovery of neonatal sepsis and determinant factors among neonates admitted in Public Hospitals of Central Gondar Zone, Northwest Ethiopia, 2021

Aim of the study: To assess the time to recovery of neonatal sepsis and determinant factors among neonates admitted in Public Hospitals of Central Gondar Zone, Northwest Ethiopia, 2021

Name of investigator: Mohammed Oumer

Name of the Organization: The University of Gondar, College of Medicine and Health Sciences, Department of Epidemiology and Biostatistics

Person to contact: This research study was reviewed, approved, and got permission from the Ethical Review Committee of the Institute of Public Health. Besides, if you want to know more information about the research and its investigators, you can contact the addresses attached below.

1. **Mr. Mohammed Oumer** (B.Sc., M.Sc., MPH in Epidemiology Student): University of Gondar

Cell phone: +251-918-809716

E-mail: mohammedoumer58@gmail.com

2. **Mr. Dessie Abebaw** (M.P.H., Assistant Professor of Epidemiology and Biostatistics)

The University of Gondar, College of Medicine and Health Sciences, Institute of Public health, Department of Epidemiology and Biostatistics

Cell phone: +251-912-480696

E-mail: dessieabebaw96@gmail.com

3. **Dr. Ashenafi Tazebew** (M.D., Associate Professor of Pediatrics and Child Health, CED of CMHS and CSH, Vice president of the University of Gondar)

The University of Gondar, College of Medicine and Health Sciences, School of Medicine, Department of Pediatrics and Child Health

Cell phone: +251-913-164722

E-mail: ashenafitazebew1@gmail.com

Annex 3: Confidentiality and informed consent statement

I am going to ask you questions about the time to recovery of neonatal sepsis, its determinant factors, and related variables before assessing your neonate. The study will be carried out through interviews, follow-up, physical assessment, and reviewing records. Your name (and other personal identifiers) will not appear on this questionnaire. All the information you provide to me will be strictly confidential and documents will be kept using password protection. All measurements will be collected without any harm to the newborn. You are not obliged to answer any questions that you do not wish to answer, and you can put an end to this interview at any time if you wish to do so. Your participation in this study does not involve any direct risk or benefit (no incentive/payment) for you or your neonate. However, it is very useful since your answers, as well as those of other participants, will help to get the average days of recovery from sepsis and its determinant factors, gives useful information for clinicians in order to care for the neonates, and provides evidence for program planners and decision-makers on treatment outcomes. Besides, it has paramount importance to curb the morbidity and mortality of neonatal sepsis.

Would you like to participate in the study?

1. Yes **(If yes, continue)**
2. No

Annex 4: Consent form

I have read and understood well the condition stated above and I can withdraw from the study at any time and I understand that there is no risk of participating and no incentive to be given when I participate in the study. Therefore, I am willing to participate in the study.

Signature____________________ Date____________

Thank you so much!

Annex 5: English data collection questionnaire

Part 1: Socio-demographic characteristics

S.No.	Questions	Choice of responses	Code
1.	Identification number		
2.	Age of the mother	…………. in years	
3.	Place of residence;<br>Kebele __ Woreda __ Zone __ Other, specify__	1. Urban<br>2. Rural	
4.	Ethnicity	1. Amhara<br>2. Oromo<br>3. Tigray<br>4. Others, specify	
5.	Marital status	1.Married<br>2.Widowed<br>3. Divorced<br>4. Single<br>5. Other, specify	
6.	Religious status	1. Orthodox<br>2. Muslim<br>3. Catholic<br>4. Protestant<br>5. Others, specify	
7.	Educational status	1. Unable to read and write<br>2. Able to read and write<br>3. Primary education/1-8<br>4. Secondary and preparatory education/9-12<br>5. Certificate and diploma holder<br>6. Degree holder and above	
8.	Educational status of the husband	1. Unable to read and write<br>2. Able to read and write<br>3. Primary education/1-8<br>4. Secondary and preparatory education/9-12<br>5. Certificate and diploma holder	

		6. Degree holder and above	
9.	Occupation	1. Housewives<br>2. Merchant<br>3. Government employee<br>4. Daily laborer<br>5. Farmer<br>6. Student<br>7. Other, specify	
10.	Monthly household income (In Birr)	_____________Birrs	
11.	Family size	___________in numbers	

Part 2: Maternal-related characteristics

**S.No.**	**Questions**	**Choice of responses**	**Code**
1.	Parity	___________.	
2.	Gravidity/ number of pregnancy	_____________.	
3.	Onset of labor	1. Spontaneous<br>2. Induced<br>3. Other, specify	
4.	Duration of labor	_____________hours	
5.	Mode of delivery	1. Spontaneous vertex delivery<br>2. Assisted instrumental delivery<br>3. Cesarean section	
6.	Place of delivery/birth	1. Home<br>2. Health institutions<br>3. Other, specify	
7.	Delivery attendant	1. Trained birth attendant/TBA<br>2. Health extension workers /HEW<br>3. Health professionals<br>4. Other, specify	
8.	Number of ANC visits	1. No<br>2. One	

		3. Two<br>4. Three<br>5. Four and above	
9.	Twin pregnancy	1. No<br>2. Yes	
10.	Obstructed labor	1. No<br>2. Yes	
11.	Foul-smelling liquor/ foul lochia	1. No<br>2. Yes	
12.	UTI/STD during pregnancy	1. No<br>2. Yes	
13.	Pregnancy-induced hypertension	1. No<br>2. Yes	
14.	Antepartum hemorrhage	1. No<br>2. Yes	
15.	Intrapartum fever	1. No<br>2. Yes	
16.	Diagnosed chorioamnionitis	1. No<br>2. Yes	
17.	Duration after the rupture of membrane	______________ in hours	
18.	Maternal infection history	1. No<br>2. Yes	
19.	Danger symptoms during pregnancy (mentioned if the answer is yes)	1. No<br>2. Yes	
20.	Presence of chronic illness	1. No<br>2. Yes, if yes please specify	
21.	Placental abnormality	1. No<br>2. Yes	

Part 3: Clinical features/presentation of neonates with sepsis

S.No.	Questions	Choice of responses	Code
1.	Have fever	1. No<br>2. Yes	
2.	Apnea	1. No<br>2. Yes	
3.	Respiratory distress	1. No<br>2. Yes	
4.	Tachycardia	1. No<br>2. Yes	
5.	Poor feeding	1. No<br>2. Yes	
6.	Dehydration	1. No<br>2. Yes	
7.	Vomiting	1. No<br>2. Yes	
8.	Lethargy	1. No<br>2. Yes	
9.	Convulsion/seizure	1. No<br>2. Yes	
10.	Irritability	1. No<br>2. Yes	
11.	Drowsiness	1. No<br>2. Yes	
12.	Bulging fontanel	1. No<br>2. Yes	
13.	Hypothermia	1. No<br>2. Yes	
14.	Capillary refilling time	1. Normal<br>2. Prolonged	
15.	Pallor	1. No<br>2. Yes	
16.	Cyanosis	1. No	

		2. Yes	
17.	Severe jaundice	1. No<br>2. Yes	
18.	Sclerema	1. No<br>2. Yes	
19.	Chest indrawing	1. No<br>2. Yes	
20.	Other signs and symptoms	If there is, specify	

Part 4: Diagnostic/laboratory test results and microbial-related characteristics

S.No.	Questions	Coding categories	Code
1.	Blood culture	1. Negative<br>2. Positive<br>3. Not applicable/Not done	
2.	Hematocrit/HCT	_______________%	
3.	Hemoglobin/Hgb	________________gm/dl	
4.	White blood cell	_________________$x10^3$ µL	
5.	Mean platelet volume	_________________ fL	
6.	Absolute neutrophil count	________________ $x10^3$ µL	
7.	Platelet count	________________ $x10^3$ µL	
8.	Red blood cell count	_________________ $x 10^6$ µL	
9.	Random blood sugar	__________________mg/dl	
10.	Platelet distribution width	_________________	
11.	C-reactive protein/CRP	_________________mg/dl	
12.	Cerebrospinal fluid (CSF) glucose	_______________	
13.	CSF protein	________________	
14.	CSF WBC $x10^6$/L	________________	
15.	CSF cellularity		
16.	Urine culture		

17.	Renal function test		
18.	Other lab results, mention if any		
19.	Abnormal radiological finding	1. No<br>2. Yes (specify, CXR, U/S, CT, MRI, other X-ray, neurosonogram)<br>3. Not applicable	
20.	Major co-morbidities	1.TB<br>2. HIV<br>3. Malaria<br>4. Diarrhea<br>5. Heart failure<br>6. Other, specify ________<br>7. Not applicable	
21.	Time of the infection onset	1. EONS<br>2. LONS	
22.	Bacterial isolates	1. Gram negative bacteria (specify, ___________________________)<br>2. Gram positive bacteria (specify, ____________________________)	
23.	Sepsis type	1. Clinical sepsis<br>2. Culture positive sepsis/confirmed sepsis	
24.	Culture	1. Bacterial isolates in blood culture<br>2. Other culture sepsis (specify)<br>3. Not applicable	

Part 5: Neonate related characteristics

S.No.	Questions	Categories/responses	Code
1.	Age of neonate at admission	________________ in hours	
2.	Sex of neonate	1. Male<br>2. Female	
3.	Birth weight	_____________gm	
4.	Gestational age at birth	_____________weeks	
5.	Admission weight	_____________gm	

6.	Temperature at admission	______________ ^{0}C	
7.	Respiratory rate at admission	______________b/m	
8.	EBF initiated within one hour	1. No<br>2. Yes	
9.	First minute APGAR score	_________________.	
10.	Fifth minute APGAR score	_________________.	
11.	Resuscitated at birth	1. No<br>2. Yes	
12.	Kept in KMC within one hour	1. No<br>2. Yes	
13.	Respiratory distress syndrome	1. No<br>2. Yes	
14.	Meconium aspiration syndrome	1. No<br>2. Yes	
15.	Pathologic/abnormal umbilical cord	1. No<br>2. Yes	
16.	Amniotic fluid abnormality	1. No<br>2. Yes	
17.	New admission	1. No<br>2. Yes	
18.	Admission date with time	____dd_____mm______yyy,<br>Time____________PM/AM	

Part 6: Health care service-related characteristics

S.No.	Questions	Choice of responses	Code
1.	Satisfied with services given for the neonate	1. No<br>2. Yes	
2.	Appropriately trained health workers in NICU	1. No<br>2. Yes	
3.	Have the NICU good quality in general	1. No	

		2. Yes	
4.	Early care-seeking at the household level	1. No<br>2. Yes 3. Not applicable	
5.	Early recognition of illness at health care level	1. No<br>2. Yes	
6.	Early initiation of treatment at health care level	1. No<br>2. Yes	
7.	Is it near the distance from your home to the nearest health facility?<br>The distance to the nearest health facility where they can be treated _________meter	1. No<br>2. Yes	
8.	Was there fast and adequate transport access from home to a health care institution	1. No<br>2. Yes	
9.	The cost of transportation from your home to this hospital made you delay in seeking treatments for your neonate	1. No<br>2. Yes	
10.	Referral, before admission to this hospital	1.Place of referral ___________<br>2. Number health facilities visited_____________<br>3. Total duration of stay ___________<br>4. Total time taken from primary care to this hospital_____________	
11.	Was there a fast referral at primary health care (if referred)	1. No<br>2. Yes	
12.	Time of visiting health facility after the neonate get sick	in hours	

Part 7: Management, complication, and outcome status characteristics

S.No.	Questions	Choice of responses	Code
1.	Naso gastric tube feeding/non-oral enteral feeding	1. No<br>2. Yes (for how many days_____)	
2.	Assisted with bag and mask/requirement of invasive ventilation	1. No<br>2. Yes	

3.	Duration of treatment	___________________hours/days	
**Medications/treatments**			
1.	Antibiotics given	1. Ampicillin<br>2. Gentamicin<br>3. Amoxicillin/Clavulanic acid<br>4. Sulphamethaxazole /trimethoprim<br>5. Tetracycline<br>6. Ceftriaxone<br>7. Cefotaxime<br>8. Ceftazidime<br>9. Ciprofloxacin<br>10. Meropenem<br>11. Vancomycin<br>12. Erythromycin<br>13. Penicillin<br>14. Clindamycin<br>15. Cloxacillin<br>16. Others, specify	
2.	Supportive care	1. No<br>2. Yes, if yes indicate what type	
3.	IV line medication or antibiotics	1. No<br>2. Yes	
4.	Blood transfusions	1. No<br>2. Yes	
5.	If there is any other type of care other than mentioned, specify which type	_____________________	
	**Complications**		
1.	Meningitis	1. No<br>2. Yes	
2.	Septic shock	1. No<br>2. Yes	
3.	Hypoxemia	1. No<br>2. Yes	
4.	Acute kidney injury/renal failure	1. No<br>2. Yes	
5.	Neurological sequelae at discharge	1. No<br>2. Yes, if yes specify	
6.	Disseminated intravascular coagulation	1. No<br>2. Yes	

7.	Respiratory failure	1. No<br>2. Yes	
8.	Cardiovascular dysfunction	1. No<br>2. Yes	
9.	Presence of organ dysfunction	1. No<br>2. Yes	
10.	Blindness and/or deafness	1. No<br>2. Yes	
11.	Infectious complications	1. No<br>2. Yes	
12.	Other complications	Specify, ____________________	
13.	Being in critical conditions/ admitted to high dependency area	1. No<br>2. Yes	
**Discharge and outcome status after admission**			
1.	Discharged as	1. Recovered<br>2. Died<br>3. Defaulted/lost follow-up<br>4. Referred<br>5.Transferred/or not-recovered	
2.	Discharge weight	____________gm	
3.	Age at discharge	hours	
4.	Length/duration of hospital stay	_________________ days	
5.	Discharge date with time	____dd_____mm______yyy,<br>Time____________PM/AM	

Date you have finished interview/data collection _______/_______/________

The time you have ended ____

Name of the data collector ______________________________ Signature ________________

Supervisor Name ______________________________________ Signature ________________

Total number of participants followed _______________________________________
