# Supplementary material for: Time to recovery of neonatal sepsis and determinant factors among neonates admitted in Public Hospitals of Central Gondar Zone, Northwest Ethiopia, 2021
Source: PLoS One. 2022 Jul 28;17(7):e0271997. doi: 10.1371/journal.pone.0271997 (PMC9374017; doi:10.1371/journal.pone.0271997)
Supplement: S3 File — (PDF) [file pone.0271997.s003.pdf]

Supporting information 3

Amharic Version Questionnaire

ስለ ጨቅላ ህፃናት ሴፕሲስ/መመርቀዝ/ የመዳን ፍጥነት እና ተያያዥ ጉዳዮች በማዕከላዊ ጎንደር ባሉ የህዝብ ሆስፒታሎች ላይ ለማጥናት የሚሞላ ቅፅ፤ 2013።

የመስሪያ ቤቱ/የሆስፒታሉ ስም --

አባሪ 1፡ መግቢያ

የጨቅላ ህፃናት ሴፕሲስ የመዳን ፍጥነት እና ተያያዥ ጉዳዮች ላይ የሚሰራ ጥናት።

የመለያ ካርድ ቁጥር -----------

ቃለ መጠይቅ የተደረገበት ቀን -----

የተጀመረበት ግዜ -------------

ስሜ ------------------------------------- እባላለሁ።

በአሁኑ ሰአት የጨቅላ ህፃናት ሴፕሲስ የመዳን ፍጥነት (የመኖር ሁኔታ) እና ተያያዥ ጉዳዮች ላይ ጥናት እየሰራን እንገኛለን። ስለዚህ የሚሰበሰበው መረጃ ለህክምና ምርመራ ጉዳይ፤ የመዳንን ፍጥነት ለመዳሰስ ወይም በ ሴፕሲስ ምክኒያት የሚመጣ የጤና ሁኔታን ለመከታተል እና ለማህበረሰባችን ከፍተኛ አስተዋጥኦ (ከመኖር ጋር የተያያዙ ችግሮችን ቀድሞ ለይቶ መፍትሄ ለመስጠት) እንደሚያበረክት ልንገልጥልዎ እንወዳለን። ለዚህ ጥናት አንች እና ልጂሽ ተመርጣችኋል።

አባሪ 2፡ መረጃን ስለመስጠት

የጥናቱ ርዕስ፡ ስለ ጨቅላ ህፃናት ሴፕሲስ የመዳን ፍጥነት (ለመዳን የሚወስደው ግዜ) እና ተያያዥ ጉዳዮች በማዕከላዊ ጎንደር ባሉ የህዝብ ሆስፒታሎች ላይ በ 2013 የሚሰራ ጥናት፡፡

የጥናቱ አላማ፡ በሴፕሲስ የተጠቁትን ጨቅላ ህፃናት የመዳን ፍጥነት እና ተያያዥ ጉዳዮችን (በማዕከላዊ ጎንደር ዞን ባሉ የህዝብ ሆስፒታሎች የሚገቡ የታመሙ ህጻናትን ያካትታል) ለመገምገም ይረዳል፡፡

የተመራማሪ ስም፡ ሙሀመድ ኡመር

የድርጅት ስም፡ ጎንደር ዩኒቨርሲቲ

በጥናቱ ጉዳይ ሰዎችን ለማነጋገር፡ ይህ ጥናት በ ህብረተሰብ ጤና አጠባበቅ የጥናት ገምጋሚ ኮሚቴ ታይቶና ተገምግሞ ፍቃድ የተሰጠው ሲሆን ከዚህም በዘለለ ስለ ተመራማሪዎች እንዲሁም ስለ ጥናቱ መረጃ ማግኘት ከፈለጉ በሚከተለው አድራሻ ማግኘት ይችላሉ፡፡

1. ሙሀመድ ኡመር (B.Sc., M.Sc., የኢፒዲሞሎጂ ተማሪ)፡ ጎንደር ዩኒቨርሲቲ

ስልክ፡ +251-918-809716

ኢሜል፡ mohammedoumer58@gmail.com

2. አቶ ደሴ አበባው (MPH፤ በ ኢፒዲሞሎጂ እና ባዮስታቲስቲክስ ረዳት ፕሮፌሰር)

ጎንደር ዩኒቨርሲቲ፤ የህክምና እና ጤና ሣይንስ ኮሌጅ፡ የህብረተሰብ ጤና አጠባበቅ ተቋም፤ ኢፒዲሞሎጂ እና ባዮስታቲስቲክስ ትምህርት ክፍል

ስልክ፡ +251-912-480696

ኢሜል፡ dessieabebaw96@gmail.com

3. **ዶ/ር አሸናፊ ታዘበው** (MD+፤ በ ፔዲያትሪክስና ህጻናት ጤና ስፔሻሊስት፤ ተባባሪ ፕሮፌሰር፤ የሆስፒታሉ ኃላፊ፣ የጎንደር ዩኒቨርሲቲ ምክትል ፕሬዘዳንት)

ጎንደር ዩኒቨርሲቲ፤ የህክምና እና ጤና ሣይንስ ኮሌጅ፡ የህክምና ት/ቤት ፤ ፔዲያትሪክስና የህጻናት ጤና ትምህርት ክፍል፡፡

ስልክ፡ +251-913-164722

ኢሜል፡ ashenafitazebew1@gmail.com

አባሪ 3፡ ሚስጥርን ስለመጠበቅ እና የመረዳት ስምምነትን ስለመግለፅ

ልጅሽን ከመመርመሬ በፊት ስለ ጨቅላ ህፃናት ሴፕሲስ የመዳን ፍጥነት፤ ተያያዥ ጉዳዮች እንዲሁም ስለ አንች አንዳንድ ጥያቄዎችን እንጠይቅሽ አለን፡፡ ጥናቱ የሚካሄደው አንቸን በመጠየቅ፤ ክትትል በማድረግ፤ ልጁን በመመርመር እና ከተመዘገበው መረጃን በመሰብሰብ ይሆናል፡፡ ስመዎትና ሌሎች ጠቋሚዎች በመጠይቁ/ቅጹ ላይ የማይጠቀስ ሲሆን የምትሰጡን ማነኛውም መረጃ ሚስጥር የተጠበቀ ነው፡፡ መረጃውም ከአጥኝው በስተቀር ሌላ እንዳይደርስበት በይለፍ ቃል የሚጠበቅ ይሆናል፡፡ ሁሉም ልኬቶች ህፃናቶችን በማይጎዳ መልኩ የሚካሄድ የሆናል፡፡ በጥናቱ መሳተፍሽ ባንችም ሆነ በልጁ/ጇ ምንም አይነት ጉዳት የማያደርስ (የተለየ የሚሰጥሽ ጥቅምም/ክፍያ የለውም) ሲሆን ያለመሳተፍም መብት እንዳለሽ ልነግርሽ እወዳለሁ፡፡ እንዲሁም ዕንድትጠየቂ የማትፈልጊውን ጥያቄ ያለመመለስ ወይም ከፈለግሽ ጥናቱን በማነኛውም ግዜ የማቋረጥ ሙሉ መብት እንዳለሽ ልነግርሽ እወዳለሁ፡፡ ነገር ግን አንችም ሆንሽ ሌሎቹ በዚህ ጥናት መሳተፋቹህና የምትሰጡት ምላሽ ህፃናቶች በ አብሬጅ በ ምን ያህል ፍጥነት እንደሚድኑ እንዲሁም በ ዚህ ፍጥነት ላይ ተፅእኖ የሚያደርሱ ፋክተሮችን የመለየት ስራ ይሰራል፡፡ ይህ መሆኑ ደግሞ ለ ጤና ባለሞያዎች ህፃናትን እንዲከባከቡ ጠቃሚ መረጃ ይሰጣል፤ ለ ፕሮግራም አቃጆችና ውሳኔ ሰጭዎች በህክምና ዉጤት ላይ መረጃ ይሰጣል፡፡ በተጨማሪም የህፃናት ሴፕሲሰ የሚያደርሰውን ሰቆቃ እና ሞት በመግታት ዙሪያ ክፍተኛ አስተዋፆኦ ይኖረዋል፡፡

በጥናቱ ላይ መሳተፍ ይፈልጋሉ !

1. አዎ **(መልሱ አዎ ከሆነ ይቀጥሉ፡፡)**
2. አልፈልግም፡፡

አባሪ 4፡ የስምምነት ፍቃድ

ከላይ የተጻፈውን መረጃ በደንብ አንብቤ ተረድቸው አለሁ፡፡ በጥናቱ ላይ መሳተፍ በኔም ሆነ በልጀ ላይ ምንም አይነት ችግር እንደማያደርስ ፤ የተለየ ጥቅማጥቅምም እንደለለው እንዲሁም በማነኛውም ሰአት ካልተመቸኝ የማቋረጥ መብት እንዳለኝ ተነግሮኛል፡፡

በመሆኑም በጥናቱ ላይ ለመሳተፍ ዝግጁ/ፍቃደኛ ነኝ ፡፡

ፊርማ -------------------- ቀን ------------------------------

ስለተሳትፎዎ እናመሰግን አለን !!!

አባሪ 5፡ የመረጃ መሰብሰቢያ ቅጽ

ክፍል 1፡ ስለ ህጻኑ እናት ሁኔታና በተያያዥ ማህበራዊ ጉዳዮች ዙሪያ የተዘጋጀ መጠይቅ/ቅጽ

ተ.ቁ	ጥያቄዎች	አማራጮች	ኮድ
1.	መለያ ቁጥር		
2.	የእናት እድሜ	-------------- በ አመት	
3.	አድራሻ/የመኖሪያ ቦታ<br>ቀበሌ-------- ወረዳ--------- ዞን-------- ሌላ ካለ ይጠቀስ-----------	1. ከተማ<br>2. ገጠር	
4.	ብሄር	1. አማራ<br>2. ኦሮሞ<br>3. ትግሬ<br>4. ሌላ ካለ ይጠቀስ	
5.	የጋብቻ ሁኔታ	1. ያገባች<br>2. ባሏ የሞተባት<br>3. የተፋታች<br>4. ገና ያላገባች<br>5. ሌላ ካለ ይጠቀስ	
6.	የሀይማኖት ሁኔታ	1. ኦርቶዶክስ<br>2. ሙስሊም<br>3. ካቶሊክ<br>4. ፕሮቴስታንት<br>5. ሌላ ካለ ይጠቀስ	
7.	የትምህርት ሁኔታ	1. ማንበብ እና መጻፍ የማትችል<br>2. ማንበብ እና መጻፍ የምትችል<br>3. የመጀመሪያ ደረጃ ትምህርት/ ከ 1-8 የተማረች<br>4. የሁለተኛና መሰናዶ ትምህርት/9-12<br>5. ሰርትፍኬትና ዲፕሎማ ያላት<br>6. ዲግሪ እና ከዛ በላይ ያላት	
8.	የአጋር/ባል የትምህርት ሁኔታ	1. ማንበብ እና መጻፍ የማይችል<br>2. ማንበብ እና መጻፍ የሚችል<br>3. የመጀመሪያ ደረጃ ትምህርት/ ከ 1-8 የተማረ<br>4. የሁለተኛና መሰናዶ ትምህርት/9-12<br>5. ሰርትፍኬትና ዲፕሎማ ያለው<br>6. ዲግሪ እና ከዛ በላይ ያለው	
9.	ስራ	1. የቤት እመቤት<br>2. ነጋዴ<br>3. የመንግስት ሰራተኛ<br>4. የቀን ሰራተኛ<br>5. ገበሬ<br>6. ተማሪ<br>7. ሌላ ካለ ይጠቀስ	
10.	የቤተሰብ የወር ገቢ. (በ ብር)	--------------------- ብር	

11.	የቤተሰብ ብዛት	-------------------------- በ ቁጥር	

ክፍል 2: ከእናትነት ጋር የተያያዘ ባህሪያት

ተ.ቁ	ጥያቄዎች	አማራጮች/ምላሾች	ኮድ
1.	ስንተኛ ልጅሽ ነዉ/ነች		
2.	ስንተኛ እርግዝናሽ ነው		
3.	የምጡ አጀማመር	1. በራሱ<br>2. በምጥ መርፌ<br>3. ሌላ ካለ ይጠቀስ	
4.	የምጡ ርዝማኔ በሰአት	--------- በሳአት	
5.	የወሊድ አይነት	1. በማህጸን አምጣ<br>2. በ መሳሪያ የታገዘ<br>3. በቀዶ ጥገና	
6.	የወለደቸበት ቦታ	1. ቤት<br>2. ጤና ተቋም<br>3. ሌላ ካለ ይጠቀስ	
7.	ያዋለዳት አካል	1. የሰለጠነ የልምድ ባለሞያ<br>2. ጤና ኢክስቴንሽን<br>3. የጤና ባለሞያ<br>4. ሌላ ካለ ይጠቀስ	
8.	ቅድመ ወሊድ ምርመራ ብዛት	1. የለኝም<br>2. አንድ<br>3. ሁለት<br>4. ሶስት<br>5. አራትና ከዛ በላይ	
9.	እርግዝናው መንትዮሽ ነበር	1. አይደለም<br>2. አዎ	
10.	ምጡ ኦብስትራክትድ ነበር	1. አይደለም<br>2. አዎ	
11.	የውልደት ፈሳሽ ሽታ ነበረው	1. አይደለም<br>2. አዎ	
12.	በርግዝና ግዜ የሽንት ትቦ ወይም የአባላዘር በሽታ ነበር	1. አይደለም<br>2. አዎ	
13.	በእርግዝና ምክኒያት የመጣ ግፊት ነበር	1. አይደለም<br>2. አዎ	
14.	ቅድመ ወሊድ ሄሞሬጅ ነበር	1. አይደለም<br>2. አዎ	
15.	በወሊድ ግዜ ትኩሳት ነበር	1. አይደለም<br>2. አዎ	
16.	ኮሪዮአምኔዎናይተስ ተገንቶባት ነበር	1. አይደለም<br>2. አዎ	
17.	የፅንሽርት ውሃ ከፈሰሰ ምን ያህል ግዜ ነበር በሰአት	ሰአት	
18.	የእናት ኢንፌክሽን/መመርቀዝ ነበር	1. አይደለም<br>2. አዎ	
19.	የእርግዝና የአደጋ ምልክቶቸ ነበሩ፡፡ ካሉ በዝርዝር ይጠቀሱ፡፡	1. አይደለም<br>2. አዎ	

20.	የግል የረጅም/ክሮኒክ ግዜ ተያያዥ በሽታ ነበር	1. አይደለም<br>2. አዎ (ካለ ይዘርዘር)	
21.	የእግዴ ልጁ ችግር ነበረበት	1. አይደለም<br>2. አዎ	

ክፍል 3: ክሊኒካል ፊውቸርስ/የበሽታው ምልክቶች

ተ.ቁ	ጥያቄዎች	አማራጮች	ኮድ
1.	ትኩሳት ነበር	1. አይደለም<br>2. አዎ	
2.	ትንፋሽ መቋረጥ ነበር/አፒኒያ	1. አይደለም<br>2. አዎ	
3.	የመተንፈሻ አካል ዲስትረስ/መቸገር ነበር	1. አይደለም<br>2. አዎ	
4.	ፈጣን የአተነፋፈስ ችግር/ታኪካርዲያ ነበር	1. አይደለም<br>2. አዎ	
5.	የአለመመገብ እግር ነበር	1. አይደለም<br>2. አዎ	
6.	የሰውነት መድረቅ ችግር ነበር	1. አይደለም<br>2. አዎ	
7.	ትውኪያ ነበር	1. አይደለም<br>2. አዎ	
8.	ሌታርጂ ነበር	1. አይደለም<br>2. አዎ	
9.	የመጣል/የመንቀጥቀጥ በሽታ ነበር	1. አይደለም<br>2. አዎ	
10.	የመቁነጥነጥ/ኢሪቴብሊቲ ችግር ነበር	1. አይደለም<br>2. አዎ	
11.	ድሮዊስነስ ችግር ነበር	1. አይደለም<br>2. አዎ	
12.	የእርግብግቢት ማበጥ ነበር	1. አይደለም<br>2. አዎ	
13.	የሙቀት መቀነስ ችግር ነበር	1. አይደለም<br>2. አዎ	
14.	ካፒለሪ ሪፊሊንግ	1. ኖርማል<br>2. የተራዘመ	
15.	ፓለር/የቆዳ ቀለም ችግር	1. አይደለም<br>2. አዎ	
16.	ሲያኖሲስ	1. አይደለም<br>2. አዎ	
17.	ከባድ ጃዎንዲስ	1. አይደለም<br>2. አዎ	
18.	ስክሌርማ	1. አይደለም<br>2. አዎ	
19.	የደረት መጎድጎድ/ቸስት እንድሮዊንግ	1. አይደለም<br>2. አዎ	
20.	ሌላ የተገኘ ምልክት ካለ ይጠቀስ	ዘርዝር----------------------	

ክፍል 4: ዲያጎነስቲክ/ላብራቶሪ ዉጤቶች እና ማይክሮቢያል ጋር የተገናኙ ባህሪያቶች

ተ.ቁ	ጥያቄዎች	አማራጮች	ኮድ
1.	የደም ካልቸር	1. ነጌቲቭ<br>2. ፖዘቲቭ<br>3. አልተሰራም	
2.	ሄማቶክሪት	%	
3.	ሄሞግሎቢን	gm/dl	
4.	ነጭ የደም ህዋስ	$x10^3$ µL	
5.	ሚን ፕላትሌት ቮሊዩም	fL	
6.	አብሶሊውት ኒውትሮፊል ካውንት	$x10^3$ µL	
7.	ፕላትሌት ካውንት	$x10^3$ µL	
8.	ቀይ የደም ህዋስ ቁጥር	$x\ 10^6$ µL	
9.	ራንደም ብለድ ሹግር	mg/dl	
10.	ፕላትሌት ዲስትርቢዩሽን ዊድዝ		
11.	ሲ ሪያክቲቭ ፕሮቲን	mg/dl	
12.	ሰረብሮ ስፓይናል ፍሉድ ግሉኮስ		
13.	ሰረብሮ ስፓይናል ፍሉድ ፕሮቲን		
14.	ሰረብሮ ስፓይናል ፍሉድ ነጭ የደም ህዋስ $x10^6$/L		
15.	ሰረብሮ ስፓይናል ፍሉድ ሴሉላሪቲ		
16.	ዩሪን ካልቸር		
17.	ሬናል ፋንክሽን ቴስት		
18.	ሌላ የላብራቶሪ ዉጤት ካለ ይጠቀስ		
19.	የራዲዎሎጂ ውጤት መዛባት አለ	1. አይደለም<br>2. አዎ (ግለጥ፤ የደረት ራጅ ፤ አልትራሳወንድ፤ ሲቲ ስካን፤ መርአይ፤ ሌላ ራጅ፤ ኒውሮሶኖግራም)<br>3. አልተላከም	
20.	ዋና ተያያዥ በሽታዎች	1. ትቢ<br>2. ኤች አይ ቪ ኤድስ<br>3. ወባ<br>4. ተቅማጥ<br>5. የልብ ድካም<br>6. ሌላ ካለ ይጠቀስ<br>7. ተያያዥ የለም	
21.	የኢንፌክሽን አጀማመር ግዜ	1. ኢርሊ ኦንሴት ሴፕሲስ<br>2. ሌት ኦንሴት ሴፕሲስ	
22.	ባክተሪያል አይዞሌትስ	1. ግራም ነጌቲቭ ባክተሪያ (ዘርዝር---)<br>2. ግራም ፖዘቲቭ ባክተሪያ (ዘርዝር--)	
23	ሰፕሲስ/መመርቀዝ አይነት	1. ክሊኒካል ሰፕሲስ<br>2. ካልቸር ፖዘቲቭ ሴፕሲስ	
24.	ካልቸር	1. ባክተርያ ደም ውስጥ ሲገኝ<br>2. ሌላ አይነት ካልቸር ሴፕሲስ<br>3. አፕልኬብል አደልም	

ክፍል 5: የጨቅላ ህፃን ጋር የተገናኙ ባህሪያቶች

ተ.ቁ	ጥያቄዎች	አማራጮች	ኮድ
1.	የጭቅላው ዕድሜ ሲገባ ወደ ሆስፒታል	------------------- በ ሰአት	
2.	የጭቅላው ጾታ	1. ወንድ<br>2. ሴት	
3.	የውልደት ክብደት	ግራም	
4.	የእርግዝና እድሜ	------------ ሳምንት	
5.	አድሚሽን ክብደት	ግራም	
6.	ቴምፐሬቸር ሲገባ	^{0}C	
7.	ሪስፓራቶሪ ሬት ሲገባ	b/m	
8.	በተወለደ አንድ ሰአት ውስጥ የእናት ወተት ብቻ ወስዷል	1. አይደለም<br>2. አዎ	
9.	የመጀመሪያ ደቂቃ አፕጋር ስኮር		
10.	አምስተኛ ደቂቃ አፕጋር ስኮር		
11.	እንደተወለደ ሪሲስተሽን ተሰርቶለታል	1. አይደለም<br>2. አዎ	
12.	በተወለደ አንድ ሰአት ውስጥ በእናት እቅፍ ውስጥ ሙቀት አግኝቷል	1. አይደለም<br>2. አዎ	
13.	የአተነፋፈስ ችግር ምልክት ታይቷል	1. አይደለም<br>2. አዎ	
14.	ሚኮኒየም ወደ መተፊሻ አካል የመሄድ ችግር ታይቷል	1. አይደለም<br>2. አዎ	
15.	እትብቱ ችግር ነበረበት	1. አይደለም<br>2. አዎ	
16.	የእንሽርት ውሀ ችግር ነበር	1. አይደለም<br>2. አዎ	
17.	የመጀመሪያ ገቢ ነው	1. አይደለም<br>2. አዎ	
18.	የገባበት ቀን ከ ሰአት ጋር	ቀን ወር አመት<br>ሰአት ፒኤም/ኤም	

ክፍል 6: ከጤና አገልግሎት ጋር የተገናኙ ባህሪያቶች

ተ.ቁ	ጥያቄዎች	አማራጮች	ኮድ
1.	ለ ህፃኑ በተሰጠው አገልግሎት ረክተዋል	1. አይደለም<br>2. አዎ	
2.	በበቂ ሁኔታ ስልጠና የወሰዱ ጤና ባለሞያዎች ክፍሉ ውስጥ ይገኛሉ	1. አይደለም<br>2. አዎ	
3.	የህፃናት ሞት ሽረት ክፍሉ ጥራት አለው በአጠቃላይ ሲታይ	1. አይደለም<br>2. አዎ	
4.	በቤት ደረጃ ፈጥኖ ህክምና አግኝቷል ወይም ሂዷል	1. አይደለም<br>2. አዎ	
5.	በ ጤና ተቋም ደረጃ ፈጥኖ በሽታው ታውቋል	1. አይደለም<br>2. አዎ	

6.	በጤና ተቋም ደረጃ ፈጥኖ ህክምና ተጅምሮለታል	1. አይደለም<br>2. አዎ	
7.	በቅርብ ርቀት የሚገኘው የጤና ተቋም ለ ቤታቹህ ቅርብ ነው ርቀቱ ምን ያህል ይሆናል ህክምና ሊሰጥ የሚችለው ኬ.ሜ.	1. አይደለም<br>2. አዎ	
8.	ፈጣን እና በቂ ትራንስፖርት አገልግሎት ከ ቤት እስከ ጤና ተቋም ይገኝ ነበር	1. አይደለም<br>2. አዎ	
9.	እስከዚህ ሆስፒታል ድረስ ያለው የትራንስፖርት ክፍያ ለህክምና እንድትዘገይ አድርጎሀል	1. አይደለም<br>2. አዎ	
10.	የሪፈራል ሁኔታ፤ ከዚህ ሆስፒታል ከመግባቱ በፊት	1. ሪፈር የተደረገበት ቦታ<br>2. ምን ያህል የጤና ተቋማት አልፏል<br>3. ምን ያህል ግዜ ቆይቷል ከመጀመሪያ ህክምና<br>4. ከመጀመሪያ ደረጃ ህክምና እስከዚህ ሆስፒታል ድረስ ምን ያህል አጠቃላይ ግዜ ወስዷል	
11.	የመጅመሪያ ደረጃ ህክምና ላይ ፈጣን የሆነ ሪፈር ነበር (ሪፈር ከሆነ)	1. አይደለም<br>2. አዎ	
12.	ህፃኑ ከታመመ እስከ ጤና ተቋም ድረስ ምን ያህል ግዜ ፈጀብሽ	በሰአት	

ክፍል 7: ማኔጅመንት፤ ኮምፕሊኬሽንስ/ውስብስብ ችግሮች እና የውጤት ሁኔታ ባህሪያቶች

ተ.ቁ	ጥያቄዎች	አማራጮች	ኮድ
1.	ምግብ በአፍንጫ ቱቦ መውሰድ	1. አይደለም<br>2. አዎ	
2.	የባግና ማስክ እርዳታ/ኢንቫዚቭ ቬንትሌሽን መፈለግ	1. አይደለም<br>2. አዎ	
3.	የህክምናው ርዝማኔ	,በሰአት/በቀናት	
ሕክምና/መዳህኒቶች			
1.	የተሰጠ አንቲባዮቲክስ	1. አሞክሳሲሊን<br>2. ጀንታማይሲን<br>3. አሞክሳሲሊን/ክላቩላኒክ አሲድ<br>4. ሰልፋሜታግዞል/ትሪሚቶፕሪም<br>5. ቴትራሳይክሊን<br>6. ሴፍትሪያግዞን<br>7. ሴፎታግዚም<br>8. ሴፍታዚዲም<br>9. ሲፕሮፍሎክሳሲን<br>10. ሜሮፒኔም<br>11. ቫንኮማይሲን<br>12. እሪትሮማይሲን<br>13. ፔኒሲሊን<br>14. ክሊንዳማይሲን<br>15. ክሎክሳሲሊን<br>16. ሌላ ካለ ይጠቀስ	
2.	ድጋፍ ሰጭ እንክብካቤ/ስፖርቲቭ ኬየር	1. የለም<br>2. አለ፤ ካለ ምን አይነት ይጠቀስ	

3.	በደም ስር/አይቪ የሚሰጡ መደህኒቶች ወይም አንቲባዮቲክስ	1. የለም<br>2. አለ	
4.	ደም ተለግሶታል	1. የለም<br>2. አለ	
5.	ከተጠቀሱት ውጭ ሌላ አይነት እንክብካቤ ካለ ይጠቀስ		
	**ኮምፕሊኬሽንስ/ውስብስብ ችግሮች**		
1.	ማጂራት ገትር/ሚኒንጃይትስ	1. የለም<br>2. አለ	
2.	ሴፕቲክ ሾክ	1. የለም<br>2. አለ	
3.	ሀይፖግሊሚያ	1. የለም<br>2. አለ	
4.	አጣዳፊ የኩላሊት ጉዳት	1. የለም<br>2. አለ	
5.	የነርቭ ችግር/ኒውሮሎጂካል ስኮየል	1. የለም<br>2. አለ፣ ካለ ይጠቀስ	
6.	ዲ. አይ. ሲ. መኖር		
7.	ከፍተኛ የአተነፋፈስ ችግር		
8.	የልብና ተያያዥ ችግሮች መኖር		
9.	ኦርጋን ዲስፋንክሽን መኖር		
10.	ማየት መሳን እና/ወይም መስማት አለመቻል	1. የለም<br>2. አለ	
11.	ኢንፌክሽየስ ኮምፕሊኬሽን መኖር		
12.	ሌሎች ውስብስብ ችግሮች ካሉ ይጠቀሱ	ዘርዝር	
13.	ከፍተኛ የሆነ የሞት ሽረት ክፍል ገብቷል/ክሪቲካል ሁኔታ ዉስጥ ነበር	1. አልገባም<br>2. ገብቷል	
	**የማስወጣት/ዲስቻርጅ እና የውጤት ሁኔታዎች**		
1.	ዲስቻርጅ ሲደረግ/ሲወጣ	1. ድኗል<br>2. ሙቷል<br>3. አቋርጧል<br>4. ወደተሸለ ተቋም ተልኳል/ሪፈር<br>5. ተዘዋውሯል/ ወይም አልዳነም	
2.	ሲወጣ የነበረው ክብደት	ግ.,ም.	
3.	ሲወጣ የነበረው እድሜ	ሰአት	
4.	ሆስፒታል የቆየበት የግዜ ርዝማኔ	ቀን	
5.	የወጣበት ቀን ከ ሰአት ጋር	ቀን ወር አመት<br>ሰአት ፒኤም/ኤም	

ቃለ መጠየቁ/ዴታ መሰብሰቡ ያለቀበት ቀን ________/________/_________

የጨረስክበት ሰአት ________________________________

የዴታ/መረጃ ሰብሳቢው ስም ____ ፊርማ _______________

የተቆጣጣሪ/ሱፐርቫይዘር ስም ____ ፊርማ _______________

ክትትል የተደረገባቸው ተሳታፊዎች ቁጥር ብዛት ____
