# Supplementary material for: Time to recovery of neonatal sepsis and determinant factors among neonates admitted in Public Hospitals of Central Gondar Zone, Northwest Ethiopia, 2021
Source: PLoS One. 2022 Jul 28;17(7):e0271997. doi: 10.1371/journal.pone.0271997 (PMC9374017; doi:10.1371/journal.pone.0271997)
Supplement: Attachment — Submitted filename: Comments to manuscript PONE-D-21-40591.pdf [file pone.0271997.s004.pdf]

The study evaluates the time of recovery neonatal sepsis and determinant factors to admitted them in a public hospital; even though is well designed, there are major point needing to be clarified

MAJOR COMMENTS

Although the work has good design and approach, the authors shown extensive description of results and tables shown and some paragraphs are repetitive and ambiguous. For example

It is recommended to try make a better and specific description of the tables, perhaps to summarize them so as not to include so many tables in the manuscript, for example, tables 2, 3, 4, 6, 7 and 8 show the frequencies and the log-rank analysis, but in the manuscript does not describe these results, only can read the percentages of each of the variables analyzed, and in page 18, only very succinctly describes the following:

"In this study, there were significant differences in survival patterns across the variables as indicated by the log-rank test estimate (and the Kaplan-Meier survival curve). Log-rank test estimate (for equality of survivor functions) of survival among septic neonates across variables has been indicated in Tables 2, 3, 4, 6, 7, and 8."

Is desirable to eliminate table 1 since on p. 8 describe the estimation of the sample size, also, table 9 has information that is not described in detail in the results. Table 5 can be omitted as it only shows frequencies and these are already described in the manuscript.

It is recommended to improve the conclusion of the manuscript in page 28; since, the abstract conclusion is more concise and better describe the findings found.

MINOR COMMENTS

In figure 1 the Y axe is incomplete, please solved it
