# Supplementary material for: Time to recovery of neonatal sepsis and determinant factors among neonates admitted in Public Hospitals of Central Gondar Zone, Northwest Ethiopia, 2021
Source: PLoS One. 2022 Jul 28;17(7):e0271997. doi: 10.1371/journal.pone.0271997 (PMC9374017; doi:10.1371/journal.pone.0271997)
Supplement: Attachment — Submitted filename: Response to the Reviewers.pdf [file pone.0271997.s005.pdf]

Point-by-Point Response Letter

To the **PLOS ONE JOURNAL**

May 10, 2022

Dear Esteemed Editors/Reviewers,

We would like to appreciate and thank the Esteemed Journal Editors (including the Academic Editor and Editors-in-Chief) and Reviewers for investing their golden time and energy to review and make critical views and give lesson-giving comments on our manuscript. This is creating an opportunity to enhance our work. We have accepted and tried to incorporate all of the comments provided. Additionally, we have responded to the issues raised by the reviewer line-by-line below. We indicated all the changes we made in the track changes feature in the body of the revised manuscript. Please find enclosed a revised manuscript entitled “Time to recovery of neonatal sepsis and determinant factors among neonates admitted in Public Hospitals of Central Gondar Zone, Northwest Ethiopia, 2021’’ Thank you very much for consideration of our manuscript for publication in the Plos One Journal!

Journal Requirements and Editor

- Dear Academic Editor (Dr. (Prof.) Colin Johnson), we revised our manuscript according to the comments given: we mentioned all of the tables in the main text (Page 15, 16, 17, 18, 19, 20 for Table 1, 2, 3, 4, 5, and 6) and described the figures (Page 21 for Fig 1 and Fig 2 in text and Page 23 for figure 3) and tables (Table 1, 2, 3, 4, 5, and 6). The axis for Figure 1 is completed. Thank you so much for your constructive recommendations.
- Our manuscript meets PLOS ONE's style requirements and the Data Availability statement is provided.

Response to the Reviewers' comments:

Corrections/revisions for Reviewer #1:

1. Reviewer #1: The study evaluates the time of recovery neonatal sepsis and determinant factors to admitted them in a public hospital; even though is well designed, there are major point needing to be clarified

   MAJOR COMMENTS

   Although the work has good design and approach, the authors shown extensive description of results and tables shown and some paragraphs are repetitive and ambiguous. For example It is recommended to try make a better and specific description of the tables, perhaps to summarize them so as not to include so many tables in the manuscript, for example, tables 2, 3, 4, 6, 7 and 8 show the frequencies and the log-rank analysis, but in the manuscript does not describe these results, only can read the percentages of each of the variables analyzed, and in page 18, only very succinctly describes the following:

   "In this study, there were significant differences in survival patterns across the variables as indicated by the log-rank test estimate (and the Kaplan-Meier survival curve). Log-rank test estimate (for equality of survivor functions) of survival among septic neonates across variables has been indicated in Tables 2, 3, 4, 6, 7, and 8."

   - Dear reviewer (Dr. (Prof.) Yelda Leal), we appreciate your constructive suggestions. We found the comments are relevant and we have addressed all issues raised above as per your request. We explained (frequencies with percentage and the log-rank test estimate, for instance) all tables in text in the result section to make the manuscript clear and understandable.
   - We describe frequencies with percentage and significant variables in the log-rank test estimate in text for each specific Table (for Tables 1, 2, 3, 4, 5, and 6). This was mentioned in the result section, Page 15, 16, 17, 18, 19, and 20. Thank you very much for your constructive suggestions and recommendations.

2. Is desirable to eliminate table 1 since on p. 8 describe the estimation of the sample size, also, table 9 has information that is not described in detail in the results. Table 5 can be omitted as it only shows frequencies and these are already described in the manuscript.

   - Dear reviewer, we found this comment is relevant and we have omitted Table 1 (please kindly see changes in the method section, sample size determination subsection, Page 8, and the end of the manuscript for Table) and Table 5 (please kindly see in the result section, diagnostic/laboratory test results subsection, Page 17 and the end of the manuscript for Table) as per your request.
   - Besides, we described Table 9 in text in the results in detail as per your request, and since the table also shows frequencies only we replaced the table with text. Please kindly see the changes made in the result section, Page 20. Thank you so much for your recommendations.

3. It is recommended to improve the conclusion of the manuscript in page 28; since, the abstract conclusion is more concise and better describe the findings found.
   - Dear reviewer, we improved the conclusion section of the manuscript, Page 30. Some concepts of abstract conclusion were mentioned also in the "Recommendation section" of the manuscript. Thank you very much for your recommendation.

4. MINOR COMMENTS
   In figure 1 the Y axe is incomplete, please solved it

   - Dear reviewer, we would like to give thanks for your helpful comments and Figure 1 has Y-axis originally but this occurred when we converted to PDF format from a word document. Currently, the issue is solved and attached.

We have carefully considered the reviewer's comments, and made changes as suggested in the “Revised Manuscript”. Hoping that our manuscript will be suitable for publication, we look forward to receiving your comments, and we can discuss further if the Editor or the Reviewer wishes.

Yours sincerely,

Mohammed Oumer (On behalf of all authors)
